# Associations between pneumonia and residential distance to livestock farms over a five-year period in a large population-based study

**DOI:** 10.1371/journal.pone.0200813

**Published:** 2018-07-17

**Authors:** Dominika A. Kalkowska, Gert Jan Boender, Lidwien A. M. Smit, Christos Baliatsas, Joris Yzermans, Dick J. J. Heederik, Thomas J. Hagenaars

**Affiliations:** 1 Wageningen Bioveterinary Research, Lelystad, The Netherlands; 2 Department of Environmental Epidemiology, Institute for Risk Assessment Sciences (IRAS), Utrecht University, Utrecht, The Netherlands; 3 NIVEL, Netherlands Institute for Health Services Research, Utrecht, The Netherlands; University of Illinois, UNITED STATES

## Abstract

In a recent study of electronic health records (EHR) of general practitioners in a livestock-dense area in The Netherlands in 2009, associations were found between residential distance to poultry farms and the occurrence of community-acquired pneumonia (CAP). In addition, in a recent cross-sectional study in 2494 adults in 2014/2015 an association between CAP and proximity to goat farms was observed. Here, we extended the 2009 EHR analyses across a wider period of time (2009–2013), a wider set of health effects, and a wider set of farm types as potential risk sources. A spatial (transmission) kernel model was used to investigate associations between proximity to farms and CAP diagnosis for the period from 2009 to 2013, obtained from EHR of in total 140,059 GP patients. Also, associations between proximity to farms and upper respiratory infections, inflammatory bowel disease, and (as a control disease) lower back pain were analysed. Farm types included as potential risk sources in these analyses were cattle, (dairy) goats, mink, poultry, sheep, and swine. The previously found association between CAP occurrence and proximity to poultry farms was confirmed across the full 5-year study period. In addition, we found an association between increased risk for pneumonia and proximity to (dairy) goat farms, again consistently across all years from 2009 to 2013. No consistent associations were found for any of the other farm types (cattle, mink, sheep and swine), nor for the other health effects considered. On average, the proximity to poultry farms corresponds to approximately 119 extra patients with CAP each year per 100,000 people in the research area, which accounts for approximately 7.2% extra cases. The population attributable risk percentage of CAP cases in the research area attributable to proximity to goat farms is approximately 5.4% over the years 2009–2013. The most probable explanation for the association of CAP with proximity to poultry farms is thought to be that particulate matter and its components are making people more susceptible to respiratory infections. The causes of the association with proximity to goat farms is still unclear. Although the 2007–2010 Q-fever epidemic in the area probably contributed Q-fever related pneumonia cases to the observed additional cases in 2009 and 2010, it cannot explain the association found in later years 2011–2013.

## Introduction

Recent analyses of human health data in a livestock-dense area in the Netherlands have found associations between community-acquired pneumonia (CAP) in humans and living close to poultry and goat farms. These analyses were part of a large research project [1,2] and its predecessor study [3]. Both were carried out in the South of The Netherlands, an area with significant public concerns about possible negative health effects of livestock farming on neighbouring residents. The concerns arose due to livestock farming in this area becoming more and more intensive in the last decades, and were amplified by the Q fever epidemic that occurred in that area between 2007 and 2010, resulting in more than 4000 notified human cases. These human cases were infected through inhalation of air contaminated with *Coxiella burnetii*, the bacterium causing Q fever, by dust and aerosols emitted from infected dairy goat farms (as well as some dairy sheep farms) [4].

Based on regression analysis of health conditions registered in >90,000 patients of 27 Dutch general practices in 2009, an increased occurrence of pneumonia was found in individuals living within 1 km of at least one poultry farm [5]. Subsequently, the same dataset was analysed by fitting a spatial kernel (or transmission kernel) model [6,7], with the added value of modelling exposure using not only the nearest farm but all farms, and avoiding the use of an arbitrary pre-set distance of e.g. 1 km. This analysis confirmed and extended the earlier results by attributing an increase in the CAP risk at any given home location to each individual poultry farm within a distance of around 1 km to the home [8].

In a recent study with a smaller population size an association between CAP and proximity to goat farms was observed; this study used data from both Electronic Health Records (EHR) and questionnaires completed by 2494 adults participating in a medical examination in 2014/2015 [9].

Here, we extend the kernel analysis of [8] across a wider period of time, a wider set of health effects, and a wider set of farm types as potential risk sources. The kernel analysis uses a spatial ‘transmission kernel’ model. Such a kernel model describes the probability that a health effect occurs in exposed individuals based on all risk contributions from all potential risk sources, with each individual contribution weighed according to the distance of the source from the exposed individual. In this way, a kernel analysis of health burden around livestock farms gives insight into the contribution of each individual livestock farm to a possible increase (or decrease) in a given health risk for local residents.

## Materials and methods

The VGO study protocol was approved by the Medical Ethical Committee of the University Medical Centre Utrecht (protocol number 13/533). The study was carried out according to Dutch legislation on privacy and the Code of Conduct for Medical Research. EHR data were available through the NIVEL Primary Care Database of the Netherlands Institute for Health Sciences Research. Patient privacy was ensured by keeping medical information and address records of patients separated at all times by using a Trusted Third Party; i.e., in terms of data that could potentially lead to the identification of individual patients, the authors had access to address data, but not in combination with medical data; i.e., in terms of data that could potentially lead to the identification of individual patients, the authors had access to address data, but not in combination with medical data. For the purpose of this study this separation was maintained by using the address records first, to create a database with distances (from residential locations to locations of potential risk sources), which were rounded off in such a way that the residential location of the anonymous individual patients could not be calculated back. This anonymized distance database was then linked to the medical information. The need to obtain informed consent from individual GP patients was waived. Dutch law allows the use of EHR for research purposes under certain conditions. According to this legislation, neither obtaining informed consent from patients nor approval by a medical ethics committee is obligatory for this type of observational studies containing no directly identifiable data (Dutch Civil Law, Article 7:458).

We investigate associations between health conditions in patients enrolled in general practice (GP) and their residential distance from farms using a spatial kernel model [6–8]. This model approach is often applied in the context of between-farm transmission of livestock diseases, where it leads to an estimated transmission kernel that describes the risk of disease transmission from an infected to a susceptible farm as a function of between-farm distance [6]. In the context of public health risks in relation to residential distance to potential risk sources the approach was first described and applied in Ref. [8]. Our analysis investigates associations between a number of different health conditions and residential distance to farms of one or more livestock categories (i.e., cattle, goats, mink, poultry, sheep, and swine) for each year from 2009 to 2013 in a livestock-dense region of the Netherlands located in the eastern part of North Brabant and the northern part of Limburg. The analysis was coded in Wolfram Mathematica v.10.4 [10].

### Data

Data were obtained from electronic health records (EHRs) of general practices in the Primary Care Database (PCD) of the Netherlands Institute for Health Services Research (NIVEL) over 2009–2013. Morbidity is registered based on the International Classification of Primary Care (ICPC) [11]. All Dutch inhabitants are obligatorily listed in a general practice and general practitioners (GPs) act as gatekeepers for specialized, secondary health care. The primary outcomes in the present study were whether patients experienced (for every year within the examined period) CAP, upper respiratory infections, inflammatory bowel disease, and lower back pain (as a ‘control’ condition). Whether a patient experienced a health effect in a given year was based on the patient having at least one episode of care for the health effect in question, considering both episodes with a starting date before as well as episodes with a starting date during the year in question. The construction of episodes is based on all records with an ICPC code in the EHR of general practices. These were available from episode records constructed by GPs themselves, morbidity and medication prescription records. ICPC codes are categorized into acute, long lasting, reversible and chronic irreversible conditions. Additional details regarding methodology are provided in [12]. Participating practices were located in small towns and villages with a population of less than 25,000. Medical records of 140,159 patients of all ages from 32 GPs who met the requirements detailed in [12] were included in the analyses. The number of GPs evaluated differed by year, ranging between 27 (2013) and 32 (2010). The number of GPs selected by year ranged between 21 (2009 and 2010) and 26 (2012 and 2013). No age restrictions were imposed on the GP patients included in the analyses (in contrast to the analyses of [5,8] that considered patients of up to 70 years old).

For calculation of the distances between homes and farms, coordinates of all farms (based on the centroid of the stables) in the study were obtained from the public provincial databases of mandatory environmental licences for keeping livestock (BVB, file livestock farms; data in 2012 were used for all years of the EHR data), and coordinates of residential locations of GP patients by geo-coding their addresses. In the analyses we included all farm locations across the provinces of North Brabant and Limburg, with the exception of goat farms with licences for less than 50 animals. For an overview of the number of farms in the study area (eastern part of North Brabant and the northern part of Limburg) we refer to Table E1 in the online supplement of [13]. Farms with multiple species (mixed farms) were categorized based on the species with largest size measured in economic size units as listed in the BVB database. ‘Goat farms’ containing less than 50 goats were excluded from our analyses in order to avoid inclusion of small non-commercial holdings. The exclusion involved seven out of 156 farms including one farm for which there was no size information.

### Spatial kernel models

As in ref. [8], the data were analysed using spatial kernel models to characterize the association between living in the vicinity of farms and health condition. In this context, a spatial kernel model describes a probability function, for individual patients experiencing a health condition in a given period, independently generated by each risk source (here: livestock farm) of a certain type [8]. This probability function is assumed to be dependent on the (omni-directional) distance between the risk source and the residential location: *p* = *p*(*r*_*ij*_), where *r*_*ij*_ is the straight-line distance between risk source *i* and the residential location of individual *j*. We use the notation *p*(*r*) = 1 − exp(−*λ*(*r*)) where *λ*(*r*) is the so-called hazard function. This hazard function is described by a small set of ‘kernel parameters’. For example, the parameterization *λ*(*r*) = *λ*_0_/(1 + (*r*/*r*_0_)^*α*^) often yields a good fit in analyses of between-farm transmission of livestock diseases [6], and it was also used in the analysis in Ref. [8]. We also introduce a distance-independent background probability *P*_*b*_ to describe a component of the health risk that is not associated with proximity to the assumed risk sources. Equivalent to this background probability, we define a distance-independent background hazard *λ*_*b*_ = ln(1 − *P*_*b*_). To test the hypothesis that a health risk is increased for individuals living in the neighbourhood of a certain type of source, the background hazard *λ*_*b*_ and the kernel parameters are estimated using maximum likelihood (ML). We calculate 95% confidence intervals for the parameters by means of the likelihood-ratio test. The likelihood function is given by:

$$L=\prod_{j\epsilon \Lambda^{NP}}p_{\mathrm{esc},j}\prod_{m\epsilon \Lambda^{P}}(1-p_{\mathrm{esc},m}).$$

Here *p*_esc,*j*_ and 1 − *p*_esc,*m*_ respectively denote the total probability that an individual *j* escapes given health effect and the probability that an individual *m* is affected by that health effect. Λ^*NP*^ is the group of all patients in the study population without the health effect, and Λ^*P*^ is the group of all patients in the study population with the health effect. From the assumed independence of the generated probability of the health effect by each individual animal husbandry it follows that the total probability *p*_esc,*j*_ of an individual *j* escaping the health effect is equal to *p*_esc,*j*_ = (1 − *P*_*b*_) ∏_*i*_*p*_esc,*ji*_, where the index *i* runs over all the livestock farms of the respective type in the study, 1 − *P*_*b*_ is the probability of an individual *j* escaping the health effect to the extent caused by the background hazard, and *p*_esc,*ji*_ = 1 − *p*(*r*_*ij*_), is the probability of an individual *j* escaping the health effect to the extent caused by livestock *i*. Using *p*(*r*) = 1 − exp(−*λ*(*r*)) we can write: *p*_esc,*ji*_ = exp(−*λ*(*r*_*ij*_)), and therefore the likelihood may be written as follows in terms of *λ*(*r*):

$$L=\prod_{j\epsilon \Lambda^{NP}}\exp\left(-\lambda_{b}-\sum_{i}\lambda (r_{ij})\right)\prod_{m\epsilon \Lambda^{P}}(1-\exp\left(-\lambda_{b}-\sum_{i}\lambda (r_{im})\right)).$$

To investigate whether a health risk is increased within a given distance range *d* of a certain type of source, one may parameterize the hazard function *λ*(*r*) as a step function:

$$\lambda (r)=\left\{ \begin{array}{c} \lambda_{0},\;r\leq d\\ \;0,\;r>d \end{array}\right.,$$

with the constant *λ*_0_ as only unknown kernel parameter. Estimating *λ*_0_ and *λ*_*b*_ from the data for a range of given radii *d* is used here as a computationally efficient approach to investigate if there are associations between a health risk and proximity of the home to potential risk sources. In case of such an association, i.e. when *λ*_0_ differs significantly from zero (alpha = 5%, likelihood-ratio test), we calculate the average ‘risk increase’ for individuals living within the distance range of one risk source of a given type as ((1 − exp(−*λ*_0_ − *λ*_*b*_))/*P*_*b*_ − 1) × 100% ≈ *λ*_0_/*P*_*b*_ × 100%, and also the ‘population attributable risk’ (PAR) as (1/*P*_tot_)(*P*_tot_ − *P*_*b*_) × 100%, where *P*_tot_ is the total proportion of the population affected by the health effect in the time period of interest. The PAR is the percentage of all cases of the relevant health effect in the analysed population which could be avoided in absence of exposure to the sources of interest.

A multivariate kernel analysis (for multiple source types) can be carried out by including a type-specific hazard function for each one of the source types considered. E.g. in case of a step-function hazard this would entail type-specific parameters *d* and *λ*_0_. In this multivariate case, we approximate the PAR of a given risk source type by taking the PAR obtained in the corresponding individual analysis for this type (an analysis that is using the same predefined distance range as used for this type in the multivariate analysis) and multiplying it by $\left(1-\exp\left(-\lambda_{0_{\mathrm{mul}}}\right)\right)/\left(1-\exp\left(-\lambda_{0_{\mathrm{ind}}}\right)\right)$, where the subscripts ‘mul’ and ‘ind’ refer to the multivariate and individual analysis, respectively.

### Univariate and multivariate analyses

The analyses are carried out in two steps. First, we perform an individual farm-type analysis for each of the different farm types considered, namely: cattle, (dairy) goats, mink, poultry, sheep, and swine. These analyses were carried out by year for all five years included in the EHR data. We investigate the possible risk increase around the farm type of interest by using the values 0.5, 1.0, 1.5, …, 5.0 km for the distance range *d*. Second, if a significant association between risk and proximity is found for one or more farm types, we perform a multivariate analysis in which all farm types are included for which a significant association was found in the individual analysis. We defined the best-fit distance range as that value amongst the values 0.5, 1.0, 1.5, …, 5.0 km for which the maximum-likelihood value was highest. In all individual analyses in which a significant association was found, a best-fit distance range *d* < 5.0 km could be identified. In the default multivariate analysis of a given year, for each farm type included in the analysis we use as value for the distance range *d* the best-fit value from the individual analysis for that farm type in that year. For completeness, we also performed multivariate analyses with (combinations of) other distance range values; this did not produce better fits.

Since false-positive (due to chance alone) associations can be expected in this type of analysis (encompassing a large number of statistical tests), we focus on those associations which were found in all years studied (2009–2013).

## Results

### CAP and proximity to livestock farms

We investigated if there were any associations between occurrence of CAP diagnosis as recorded in the EHR data, and proximity of the home to livestock farms. The main results are shown in Table 1. Consistently across all years from 2009 to 2013, we found an association between increased risk for pneumonia and proximity to poultry farms as well as an association between increased risk for pneumonia and proximity to (dairy) goat farms. Except for the case of poultry farms in 2011, the associations are found for at least two values of the distance range *d*, with the risk increase being larger for the shorter value(s) of the distance range. For poultry farms a distance range of 1 or 1.5 km provides the best description of the data, for goat farms there is a consistent increase in risk for ranges up to 2 km or more, with a 1.5 or 2 km range giving the best description of the data. Univariate kernel analysis over the period 2009–2013 indicates that CAP risk is increased across a time-average range of about 1 km for poultry and about 1.5 km for goats. No consistent associations are found for any of the other four farm types considered (for detailed results see S1 Table). The associations for poultry and goats are found initially in the univariate kernel analysis and remain present in the multivariate analysis for all the years from 2009 to 2013. Associations found in an individual analysis for cattle (in four of the five years) largely disappear in the multivariate analysis. From Table 1 we see that the best-fit range for poultry varies between 1 and 1.5 km, and the PAR varies between 3.1 (2013) and 9.6 percent (2010), with an approximate average of 7.2%. Within a distance range of 1 km around an average poultry farm the CAP risk is increased by between 3.7% (2013) and 15.9% (2010). Around dairy goat farms the risk increase (now within a distance range of 1.5 or 2 km) varies between 12.3% (2013) and 52.1% (2009). The PAR varies between 2.7% and 10.1%, with an approximate average of 5.4%.

**Table 1:** Multivariate kernel analyses.

	2009		2010		2011		2012		2013	
CAP cases per 1000	17.3		15.4		15.2		16.8		18.0	
Farm type	Goats	Poultry	Goats	Poultry	Goats	Poultry	Goats	Poultry	Goats	Poultry
Distance range in km	2	1	1.5	1	1.5	1	1.5	1.5	2	1
Risk increase (%)	52.1	14.8	13.6	15.9	31.7	14.3	34.0	5.6	12.3	3.7
PAR (%)	10.1	7.9	2.7	9.6	5.0	8.2	5.0	7.3	4.0	3.1

Results of multivariate kernel analyses for pneumonia by year. Only the outcomes for poultry and goats, for which consistent associations were found, are listed; for further detail see S1 Table. ‘CAP cases per 1000’ is the occurrence of CAP (registered with the GP) for each year. ‘Distance range in km’ is the distance range *d* for which the best-fit in the individual analysis for the given year was found. ‘Risk increase’ denotes the average increase of the CAP risk for individuals living within the distance range of one farm of the given type, and is calculated as described in the Materials and Methods section.

To determine whether or not the observed association between increased risk for pneumonia and proximity to goat farms arise solely due to farms that have been previously infected with Q-fever, we performed an additional kernel analysis differentiating between goat farms according to historic Q-fever status. Here we grouped farms into those that never throughout the period 2006–2013 had a Q-fever infected status, and those that did have the Q-fever infected status some time during that period. The latter are further divided into infected farms with abortions and infected farms without (reported) abortions (only bulk-milk positive). We also included poultry farms into the analysis. In four of the five analysed years, we found an association between pneumonia and proximity to goat farms that never had the Q-fever infected status (see S2 Table). This shows that the association between increased risk for pneumonia and proximity to goat farms is not solely attributable to farms that have been previously infected with Q fever.

In order to determine whether the associations found were not attributable to a local effect (for example, of one general practice), we additionally analysed the data set stratified by the GP instead of by year. These were univariate analyses taking together all years for which the respective GP was included into the EHR dataset between 2009 and 2013 according to the registration quality requirements. Around poultry farms, we found a significant CAP risk increase in nine out of the 32 practices; for five of the nine practices a significant association occurs at more than one distance range considered (results not shown). No obvious common properties were observed across these practices. For pneumonia around goat farms, we find a significant increase in 12 out of the 32 practices; for three out of the 12 practices a significant association occurs at more than one distance range considered. Again, no obvious common properties were observed across these practices. The fact that we found these significant associations despite the smaller population sizes in analyses stratified by GP provides evidence that the associations found in the overall analyses are robust, and reflect underlying patterns across at least a substantial geographical part of the study area.

### Other health risks and proximity to livestock farms

In univariate kernel analyses for upper respiratory tract infections, no consistent associations with proximity to livestock were found. Although an earlier regression analysis found an association between inflammatory bowel diseases and proximity to mink farms [12], we did not find any consistent associations within this study. We also did not find consistent associations in kernel analyses for the ‘control’ condition of lower back pain.

## Discussion and conclusions

In this paper we extended previous analyses of associations between community-acquired pneumonia and proximity to livestock farms across a wider period of time (2009–2013), a wider set of health effects, and a wider set of livestock farm types. In these analyses we used a recently developed kernel model approach, in which a spatial kernel models the distance dependent per-farm contribution to the health effect considered [8]. We found an association between the presence of poultry at distances up to around 1 kilometre and an increased risk of pneumonia. We also found an association between the presence of (dairy) goat farms within a distance range of 1.5 to 2 kilometres and an increased risk of pneumonia. Both associations were found to be consistent across all years from 2009 to 2013. No consistent associations were found for any other species considered (cattle, mink, sheep and swine), nor for any of the other health effects considered (upper respiratory tract infection, inflammatory bowel disease and lower back pain).

The association between CAP risk and proximity to poultry farms confirms and extends earlier results based on EHR data for the year 2009 [5,8]. Regarding the association between CAP risk and proximity to (dairy) goat farms, a similar association was also found in the much smaller sub-population of patients participating to a medical study [9]: Residents within 2,000m of a farm with at least 50 goats had a distance-dependent increased risk of pneumonia.

This study used a kernel analysis approach with step-function kernels to identify spatial associations between health risk and potential risk sources. In the previous analysis of EHR data over the year 2009 reported by [8], we used a more general kernel shape with three parameters, and in which the distance range parameter is not set but estimated. There the best fit to the data based on Akaike’s information criterion (AIC), describing the association between CAP risk and proximity to poultry farms, ‘reduced’ the kernel function to a step function (with a distance range of 1.15 km). This phenomenon was also observed in explorative univariate kernel analyses of the present EHR dataset with poultry farms as risk sources for the years 2009 up until 2013. Also for a set of further alternative kernel parameterizations designed to give even more flexibility, a step function occurred as best fit model based on AIC. The best-fit range in which the risk is increased differs somewhat between the years and moves between values of less than 1 to about 2 km.

A limitation of this study is that it was not attempted to adjust our analyses for possible confounders such as age, gender and occupational exposure; taking these factors into account in the framework of a kernel analysis would multiply the number of parameters to be estimated and thus computational intensity. Possible age effects were investigated in the earlier work [8] by carrying out separate kernel analyses for children and adults, and there it was found that both subpopulations showed similar excess CAP risks at close proximity from poultry farms.

Our kernel model approach assumes rotational symmetry (isotropy) and thus assesses whether health risks are related to residential distance to livestock farms irrespective of direction. Clearly, for situations in which the actual risk pattern is strongly anisotropic this approach may fail to identify risk increases. In order to assess specific anisotropic candidate mechanisms underlying certain health effects, anisotropic mechanistic modelling may be used: e.g. plume models that take into account historic data on local wind directions to model wind-borne dispersal of risk particles [14].

The underlying causes of the associations found are still to be identified. Regarding the association between CAP and poultry, a plausible explanation is the particulate matter (PM) emitted from poultry farms. There are strong indications in the literature that exposure to PM and its components such as endotoxins increases the susceptibility to CAP [15]. It is therefore conceivable that this is also the case for exposure to fine dust emitted from poultry farms. In the Netherlands and countries with similar poultry housing systems, poultry farms are known to be significant contributors to air pollution [16], particularly after conventional cage systems for laying hens were replaced by floor systems and aviary systems for animal welfare reasons [17]. As an alternative or additional mechanism, a role for specific pathogens originating from poultry cannot be ruled out, although this is not supported by a recent study of CAP in hospital patients and controls in the province of North Brabant in 2008 and 2009 [18]. In this study, no relationship was found between specific causes of pneumonia and proximity of the home to poultry farming. Nor was any association found between pneumonia caused by *Chlamydia psittaci* (a zoonotic pathogen in birds) and proximity to poultry farms. In line with the hypothesis of PM or endotoxin as an underlying cause, a further study in the same population of hospital patients as in [18] suggested that patients with CAP living at a close distance from poultry farms have a different oropharyngeal microbiota composition, with a higher abundance of *Streptococcus pneumoniae* in patients living near poultry farms [8]. This points to the possibility that high exposure to dust may lead to shifts in the microbiome that in turn, as evidenced in animal and *in vitro* experiments [19,20] may enhance the CAP risk. As stated in Ref. [8], to obtain more solid evidence for this possibility, the increased abundance of *S*. *pneumoniae* near farms would need to be replicated in larger, independent studies.

Regarding the association between CAP and dairy goat farms, no single possible explanation is standing out at this moment. Under normal circumstances the fine dust emission from dairy goat farms (for recent measurements see [21]) is small in comparison to that of e.g. poultry farms in The Netherlands, such that an explanation through PM emission enhancing susceptibility to CAP seems less plausible than a potential role for micro-organisms. Regarding a potential role for *Coxiella burnetii*, the bacteria causing Q-fever, the following remarks are in order: In view of the Q-fever epidemic that occurred in the study area between 2007 and 2010, and the fact that Q-fever may often present as pneumonia, the question arises whether the association found may be directly or indirectly due (or partly due) to Q-fever infection. This question is further motivated by the observation that the CAP risk increase is relatively high in 2009, coinciding with a high-occurrence year in the Q-fever epidemic. However, in an analysis differentiating between goat farms according to historic Q-fever status and detailed in the Supplementary Material, we found an association between CAP and proximity to goat farms that never had the Q-fever infected status (in addition to an association between CAP and proximity to goat farms with historic Q-fever infected status). This shows that the association between increased risk for pneumonia and proximity to goat farms is not solely attributable to farms that have been previously infected with Q-fever. In line with this evidence, it has been observed in the medical study that individuals with pneumonia were not more often seropositive for *Coxiella burnetii* [9].

In the Netherlands, public health concerns are currently playing an important role in the policy making around livestock farming. In 2017, these concerns, including the enhanced CAP risk in the vicinity of poultry farms, led the Dutch government to plan a reduction of the emissions from poultry houses by 50% over a period of ten years.

## Supporting information

S1 Table Detailed results of kernel analyses. Results for individual and multivariate kernel analyses for CAP around different farm types by year. ‘Sign. extent (km)’ denotes the lowest and highest values of the distance range *d* (from the set of values of 0.5, 1.0, …, 5.0 km) for which a significant (p<0.05, likelihood-ratio test) risk increase was found (individual analyses).
(DOCX)

S2 Table Analysis stratifying by Q fever status. Results of multivariate kernel analysis for pneumonia around goat farms, distinguishing between farms according to Q fever status in the period 2006–2013. Q-abortion: farms that had infected status with abortions; Q-bulk milk: farms that had infected status without (reported) abortions; Q-negative: farms that never had infected status in the period considered. +: Association between increased risk and proximity to given farm type (p<0.05, likelihood-ratio test); −: No association.
(DOCX)

S1 File Mathematica code for the analyses. (PDF)
