# Supplementary material for: Associations between pneumonia and residential distance to livestock farms over a five-year period in a large population-based study
Source: PLoS One. 2018 Jul 17;13(7):e0200813. doi: 10.1371/journal.pone.0200813 (PMC6049940; doi:10.1371/journal.pone.0200813)
Supplement: S1 Table — Results for individual and multivariate kernel analyses for CAP around different farm types by year. ‘Sign. extent (km)’ denotes the lowest and highest values of the distance range d (from the set of values of 0.5, 1.0, …, 5.0 km) for which a significant (p<0.05, likelihood-ratio test) risk increase was found (individual analyses). (DOCX) [file pone.0200813.s001.docx]

| 2009 | number of cases per 1000: 17.3 | | | | | | | | | | |
| --- | --- | --- | --- | --- | --- | --- | --- | --- | --- | --- | --- |
| *Individual analysis* | Cattle | Goats | Mink | Poultry | Sheep | Swine | *Multivariate analysis* | Cattle | Goats | Poultry | Sheep |
| Best-fit $d$(km) | 1.5 | 2 | - | 1 | 5 | - | $d$(km) | 1.5 | 2 | 1 | 5 |
| *λ_0_* | 0.0002 | 0.0087 | - | 0.0036 | 0.0008 | - | *λ_0_* | 4.5E-8 | 0.0072 | 0.0020 | 0.0001 |
| *λ_b_* | 0.0153 | 0.0153 | - | 0.0150 | 0.0144 | - | *λ_b_* | 0.0139 |  |  |  |
| Risk increase (%) | 1.6 | 57.4 | - | 23.9 | 5.7 | - | Risk increase (%) | 0.0 | 52.1 | 14.8 | 0.9 |
| PAR (%) | 12.2 | 12.2 | - | 13.9 | 17.5 | - | PAR (%) | 0.0 | 10.1 | 7.9 | 2.7 |
| Sign. extent (km) | {1.,5.} | {0.5,5.} | - | {0.5,5.} | {5.,5.} | - |  |  |  |  |  |
| **2010** | **number of cases per 1000: 15.4** | | | | | | | | | | |
| *Individual analysis* | Cattle | Goats | Mink | Poultry | Sheep | Swine | *Multivariate analysis* |  | Goats | Poultry |  |
| Best-fit $d$(km) | - | 1.5 | - | 1 | - | - | $d$(km) |  | 1.5 | 1 |  |
| *λ_0_* | - | 0.0028 | - | 0.0024 | - | - | *λ_0_* |  | 0.0018 | 0.0022 |  |
| *λ_b_* | - | 0.0148 | - | 0.0138 | - | - | *λ_b_* |  | 0.0136 |  |  |
| Risk increase (%) | - | 18.9% | - | 17.4% | - | - | Risk increase (%) |  | 13.6% | 15.9% |  |
| PAR (%) | - | 4.1% | - | 10.7% | - | - | PAR (%) |  | 2.7% | 9.6% |  |
| Sign. extent (km) | - | {1.5,2.} | - | {0.5,5.} | - | - |  |  |  |  |  |
| **2011** | **number of cases per 1000: 15.2** | | | | | | | | | | |
| *Individual analysis* | Cattle | Goats | Mink | Poultry | Sheep | Swine | *Multivariate analysis* | Cattle | Goats | Poultry |  |
| Best-fit $d$(km) | 4.5 | 1.5 | - | 1 | - | - | $d$(km) | 4.5 | 1.5 | 1 |  |
| *λ_0_* | 0.0000 | 0.0050 | - | 0.0023 | - | - | *λ_0_* | 1.4E-5 | 0.0039 | 0.0017 |  |
| *λ_b_* | 0.0116 | 0.0144 | - | 0.0137 | - | - | *λ_b_* | 0.0122 |  |  |  |
| Risk increase (%) | 0.4% | 35.0% | - | 17.2% | - | - | Risk increase (%) | 0.1% | 31.7% | 14.3% |  |
| PAR (%) | 24.1% | 6.4% | - | 11.0% | - | - | PAR (%) | 7.5% | 5.0% | 8.2% |  |
| Sign. extent (km) | {0.5,5.} | {1.,5.} | - | {1.,3.5} | - | - |  |  |  |  |  |
| **2012** | **number of cases per 1000: 17.3** | | | | | | | | | | |
| *Individual analysis* | Cattle | Goats | Mink | Poultry | Sheep | Swine | *Multivariate analysis* | Cattle | Goats | Poultry | Sheep |
| Best-fit $d$(km) | 4.5 | 1.5 | - | 1.5 | 4.5 | - | $d$(km) | 4.5 | 1.5 | 1.5 | 4.5 |
| *λ_0_* | 0.0001 | 0.0054 | - | 0.0012 | 0.0006 | - | *λ_0_* | 2.3E-5 | 0.0042 | 0.0007 | 0.0002 |
| *λ_b_* | 0.0124 | 0.0158 | - | 0.0147 | 0.0152 | - | *λ_b_* | 0.0125 |  |  |  |
| Risk increase (%) | 0.5% | 34.7% | - | 8.4% | 3.9% | - | Risk increase (%) | 0.2% | 34.0% | 5.6% | 1.6% |
| PAR (%) | 26.8% | 6.4% | - | 12.9% | 10.3% | - | PAR (%) | 10.8% | 5.0% | 7.3% | 3.5% |
| Sign. extent (km) | {1.,5.} | {1.,5.} | - | {0.5,4.} | {4.5,5.} | - |  |  |  |  |  |
| **2013** | **number of cases per 1000: 18.0** | | | | | | | | | | |
| *Individual analysis* | Cattle | Goats | Mink | Poultry | Sheep | Swine | *Multivariate analysis* | Cattle | Goats | Poultry |  |
| Best-fit $d$(km) | 0.5 | 2 | - | 1 | - | - | $d$(km) | 0.5 | 2 | 1 |  |
| *λ_0_* | 0.0010 | 0.0024 | - | 0.0010 | - | - | *λ_0_* | 0.0007 | 0.0020 | 0.0006 |  |
| *λ_b_* | 0.0172 | 0.0172 | - | 0.0171 | - | - | *λ_b_* | 0.0163 |  |  |  |
| Risk increase (%) | 5.7% | 14.3% | - | 6.0% | - | - | Risk increase (%) | 4.4% | 12.3% | 3.7% |  |
| PAR (%) | 5.1% | 4.9% | - | 5.3% | - | - | PAR (%) | 3.7% | 4.0% | 3.1% |  |
| Sign. extent (km) | {0.5,0.5} | {1.,5.} | - | {1.,3.} | - | - |  |  |  |  |  |
