# Supplementary material for: Associations between pneumonia and residential distance to livestock farms over a five-year period in a large population-based study
Source: PLoS One. 2018 Jul 17;13(7):e0200813. doi: 10.1371/journal.pone.0200813 (PMC6049940; doi:10.1371/journal.pone.0200813)
Supplement: S2 Table — Results of multivariate kernel analysis for pneumonia around goat farms, distinguishing between farms according to Q fever status in the period 2006–2013. Q-abortion: farms that had infected status with abortions; Q-bulk milk: farms that had infected status without (reported) abortions; Q-negative: farms that never had infected status in the period considered. +: Association between increased risk and proximity to given farm type (p<0.05, likelihood-ratio test); −: No association. (DOCX) [file pone.0200813.s002.docx]

|  | Q-abortions | Q-bulk milk | Q-negative | Poultry |
| --- | --- | --- | --- | --- |
| 2009 | + | + | + | + |
| 2010 | − | − | + | + |
| 2011 | + | + | + | + |
| 2012 | + | + | + | + |
| 2013 | + | + | − | + |
