# Supplementary material for: Associations between pneumonia and residential distance to livestock farms over a five-year period in a large population-based study
Source: PLoS One. 2018 Jul 17;13(7):e0200813. doi: 10.1371/journal.pone.0200813 (PMC6049940; doi:10.1371/journal.pone.0200813)
Supplement: S1 File — (PDF) [file pone.0200813.s003.pdf]

Purpose-written kernel analysis code in *Mathematica*

Kalkowska et al. ‘Associations between pneumonia and residential distance to livestock farms over a five-year period in a large population-based study’
S1 File

Subroutines

```
Needs["JLink`"];
ReinstallJava[JVMArguments → "-Xmx16g"];
toPA = Developer`ToPackedArray;
```

Import data

```
(* Disease / syndrom name used in data files *)
name = "Pneumonia";

(* The data is stratified by year and farm type *)
dataYears = {"2009", "2010", "2011", "2012", "2013"};
nDataYears = Length[dataYears];
farmTypes = {"Cattle", "Goats", "Mink", "Poultry", "Sheep", "Swine"};
nFarmTypes = Length[farmTypes];

(* Choose for which years and farm types to perform the calculations *)
years = {1, 2, 3, 4, 5};
farms = {1, 2, 3, 4, 5, 6};

(* Optimization parameters starting points *)
z00 = Table[i, {i, 0.5, 5.0, 0.5}]; (* distance z0 *)
p00 = 0.004; (* level of impact p0 *)
c00 = 0.014 ;(* background probability p1 *)
```

Optimisation

```
startSim = AbsoluteTime[];
```

General functions

```
(* Create a frame for the results by year(rows) and farm type (column) *)
printOut[x_] := Module[{table},
    table = Table[{}, {year, nDataYears + 1}, {farm, nFarmTypes + 1}];
    table[[1]] = Flatten[{{"Year"}, farmTypes}];
    table[[All, 1]] = Flatten[{{"Year"}, dataYears}];
    table[[Range[2, nDataYears + 1], Range[2, nFarmTypes + 1]]] = x;
    Return[table]
  ];

(* Calculate AIC *)
aic[l_, k_] := -2 * Log[l] + 2 * k

(* Kernel function for single farm type *)
λ[p0_, z0_, 0.] := 0;
λ[p0_, z0_, 100.] := 0;
λ[p0_, z0_, z_] := p0 * UnitStep[z0 - z];
SetAttributes[λ, Listable];

(* Round decimals in distances higher than rmax *)
rmax = 10.;
radmaxfun[r_] := r /; r ≤ rmax;
radmaxfun[r_] := 100. /; rmax < r
SetAttributes[radmaxfun, Listable];
```

Maximum likelihood per farm type per data year

```
(* Constant *)
nParametersConst = 1;
maxLogLikeConst = Table[{}, {year, nDataYears}, {farm, nFarmTypes}];

(* Block *)
nParametersBlock = 2;
degriesOfFreedom = Abs[nParametersConst - nParametersBlock];
logLikeRatio =
  Table[ "-", {z, Length[z00]}, {year, nDataYears}, {farm, nFarmTypes}];
significantFarmTypes = Table[0, {z, Length[z00]},
    {year, nDataYears}, {farm, nFarmTypes}];
maxLogLikeBlock = Table[{}, {z, Length[z00]},
    {year, nDataYears}, {farm, nFarmTypes}];
maxLogLikeBlockCI = Table[{}, {z, Length[z00]},
    {year, nDataYears}, {farm, nFarmTypes}, {i, 2}];
z0min = Table["-", {year, nDataYears}, {farm, nFarmTypes}];
z0minPos = Table["-", {year, nDataYears}, {farm, nFarmTypes}];
z0best = Table["-", {year, nDataYears}, {farm, nFarmTypes}];
z0bestPos = Table["-", {year, nDataYears}, {farm, nFarmTypes}];
z0max = Table["-", {year, nDataYears}, {farm, nFarmTypes}];
z0maxPos = Table["-", {year, nDataYears}, {farm, nFarmTypes}];

(* Multi *)
maxLogLikeBlockMulti = Table[{}, {year, nDataYears}];
maxLogLikeBlockMultiCI = Table[{}, {year, nDataYears}];
```

```
Do[
  (* IMPORT "case" and "non-case" data from input files *)
  nonCaseDistances = Table[{}, {farm, nFarmTypes}];
  caseDistances = Table[{}, {farm, nFarmTypes}];
  Do[
   Print["Reading in ", dataYears[[year]],
    " data of patients in the vicinity of ", farmTypes[[farm]], "..."];
   If[FileByteCount[FileNameJoin[{NotebookDirectory[],
        "data", ToString[dataYears[[year]]], "Escape" <> name <>
         farmTypes[[farm]] <> dataYears[[year]] <> ".txt"}]] < 4 500 000 000,
    nonCaseDistances[[farm]] = toPA@Import[FileNameJoin[
        {NotebookDirectory[], "data", ToString[dataYears[[year]]], "Escape" <>
          name <> farmTypes[[farm]] <> dataYears[[year]] <> ".txt"}], "Table"]
    ,
    nonCaseDistances[[farm]] =
     toPA@ReadList[FileNameJoin[{NotebookDirectory[],
           "data", ToString[dataYears[[year]]], "Escape" <> name <>
            farmTypes[[farm]] <> dataYears[[year]] <> ".txt"}],
         Record] // StringSplit // ToExpression
   ];
   caseDistances[[farm]] =
    toPA@Import[FileNameJoin[{NotebookDirectory[], "data",
        ToString[dataYears[[year]]], "Cases" <> name <>
         farmTypes[[farm]] <> dataYears[[year]] <> ".txt"}], "Table"]
   , {farm, farms}];

  (* CONSTANT FUNCTION MAXIMUM LIKELIHOOD PER FARM TYPE IN GIVEN YEAR *)

  (* Probability of infection *)
  pInfConst := Module[{},
    Return[-2 * Log[1 - Exp[- (p1)]]]
   ];

  (* Probability of escape *)
  pEscConst := Module[{},
    Return[2 * (p1)]
   ];

  (* Calculate MLL(const) *)
  Do[
   Print["Calculating MLL(Const) for ", dataYears[[year]],
    " data of patients in the vicinity of ", farmTypes[[farm]], "..."];
   logLikeInf = Length[caseDistances[[farm]]] * pInfConst;
   logLikeEsc = Length[nonCaseDistances[[farm]]] * pEscConst;
   maxLogLikeConst[[year, farm]] =
    FindMinimum[{(logLikeInf + logLikeEsc), p1 > 0}, {{p1, c00}},
     AccuracyGoal → 5, PrecisionGoal → 5, Method -> "InteriorPoint"];
   , {farm, farms}];

  (* Save MLL(const) *)
  saveFile = {Join[{"Farm"}, Table[farmTypes[[farm]], {farm, farms}]],
    Join[{"z0"}, Table[0., {farm, farms}]],
    Join[{"p0"}, Table[0., {farm, farms}]],
```

```
  Join[{"p1"}, Table[maxLogLikeConst[[year, farm, 2, 1, 2]], {farm, farms}]],
  Join[{"mll"}, Table[maxLogLikeConst[[year, farm, 1]], {farm, farms}]]};
Print[Column[{"Estimates of Const MLL in " <>
    ToString[dataYears[[year]]] <> ":", saveFile // Grid}]];
Export[FileNameJoin[{NotebookDirectory[], "results",
   ToString[dataYears[[year]]],
   dataYears[[year]] <> "-Const-MLL.txt"}], saveFile, "TSV"];

(* BLOCK FUNCTION MAXIMUM LIKELIHOOD PER FARM TYPE IN GIVEN YEAR *)

(* Probability of infection *)
pInfBlock[case_] := Module[{rij, aantal},
  {rij, aantal} = Transpose[Tally[radmaxfun[case]]];
  Return[-2 * Log[1 - Exp[- (Total[(aantal * λ[p0, z0, rij])] + p1)]]];
 ];

(* Probability of escape *)
pEscBlock[noncase_] := Module[{rij, aantal},
  {rij, aantal} = Transpose[Tally[radmaxfun[noncase]]];
  Return[2 * (Total[(aantal * λ[p0, z0, rij])] + p1)];
 ];

(* Optimization and CI functions *)
kernelfun[z_?NumericQ] := FindMinimum[ ((logLikeInf + logLikeEsc) /. z0 → z),
  {{p0, p00}, {p1, c00}}, AccuracyGoal → 4, PrecisionGoal → 4,
  Method -> "InteriorPoint"(*,StepMonitor:→Print[{p0,p1}]*)];
CIfun1[p_?NumericQ, z_?NumericQ] :=
 FindMinimum[ ((logLikeInf + logLikeEsc) /. {p0 → p , z0 → z}), {{p1, p1CIini}},
   AccuracyGoal → 4, PrecisionGoal → 4, Method -> "InteriorPoint"][[1]];
CIfun0[p_?NumericQ, z_?NumericQ] :=
 FindMinimum[ ((logLikeInf + logLikeEsc) /. {p1 → p, z0 → z}), {{p0, p0CIini}},
   AccuracyGoal → 4, PrecisionGoal → 4, Method -> "InteriorPoint"][[1]];

(* Calculate MLL(block) *)
Do[
 (* MLL *)
 logLikeInf = Sum[pInfBlock[i], {i, caseDistances[[farm]]}];
 logLikeEsc = Sum[pEscBlock[i], {i, nonCaseDistances[[farm]]}];
 Do[
  Print["Calculating MLL(Block) for ",
   dataYears[[year]], " data of patients in the vicinity of ",
   farmTypes[[farm]], "... z0 = ", z00[[d]]];
  maxLogLikeBlock[[d, year, farm]] = Check[kernelfun[z00[[d]]], {}];
  maxLogLikeBlock[[d, year, farm]] =
   If[NumericQ[maxLogLikeBlock[[d, year, farm, 1]]],
    maxLogLikeBlock[[d, year, farm]], {}];
  logLikeRatio[[d, year, farm]] = If[maxLogLikeBlock[[d, year, farm]] ≠ {} ,
    1.0 - CDF[ChiSquareDistribution[degriesOfFreedom], Abs[maxLogLikeConst[[
         year, farm, 1]] - maxLogLikeBlock[[d, year, farm, 1]]]]], 1];
  significantFarmTypes[[d, year, farm]] =
   If[logLikeRatio[[d, year, farm]] ≤ 0.05, 1, 0];
  Print["mll = ", maxLogLikeBlock[[d, year, farm]], ",  llr = ",
   logLikeRatio[[d, year, farm]] , ", significant = ",
```

```
    significantFarmTypes[[d, year, farm]]];
   , {d, Length[z00]}
  ];
  (* Look only at significant results *)
  If[Total[significantFarmTypes[[All, year, farm]]] > 0,
   (* list of significant distances for given year and farm type *)
   sd =
    Flatten[Position[significantFarmTypes[[All, year, farm]], _?Positive]];
   z0minPos[[year, farm]] = FirstPosition[
      significantFarmTypes[[All, year, farm]], 1][[1]];
   z0min[[year, farm]] = z00[[z0minPos[[year, farm]]]];
   z0best[[year, farm]] =
    If[Total[significantFarmTypes[[All, year, farm]]] == 1, z00[[
      Flatten[Position[significantFarmTypes[[All, year, farm]], 1]][[1]]]],
     z00[[sd[[Flatten[Position[maxLogLikeBlock[[sd, year, farm, 1]],
           Min[maxLogLikeBlock[[sd, year, farm, 1]]]]][[1]]]]]]];
   z0bestPos[[year, farm]] = Flatten[Position[z00, z0best[[year, farm]]]][[
     1]];
   z0maxPos[[year, farm]] = Length[z00] - FirstPosition[
       Reverse[significantFarmTypes[[All, year, farm]]], 1][[1]] + 1;
   z0max[[year, farm]] = z00[[z0maxPos[[year, farm]]]];
   Print["z0: ", z0best[[year, farm]], " [" ,
    z0min[[year, farm]], "," , z0max[[year, farm]], "]"];
   Do[
    Print["Calculating CI(Block) for ", dataYears[[year]],
     " data of patients in the vicinity of ", farmTypes[[farm]], "..."];
    (* Get initial conditions for the variables for CI calculations *)
    p0CIini = maxLogLikeBlock[[d, year, farm, 2, 1, 2]];
    p1CIini = maxLogLikeBlock[[d, year, farm, 2, 2, 2]];
    ml1 = maxLogLikeBlock[[d, year, farm, 1]] + 3.84;
    (* Calculate CIs *)
    maxLogLikeBlockCI[[d, year, farm, 1]] =
     {"p0", p0 /. maxLogLikeBlock[[d, year, farm, 2]],
      min /. FindRoot[CIfun1[min, z00[[d]]] == ml1,
        {min, 0.7 p0CIini, p0CIini}, AccuracyGoal → 4, PrecisionGoal → 4],
      max /. FindRoot[CIfun1[max, z00[[d]]] == ml1, {max, p0CIini, 1.5 * p0CIini},
        AccuracyGoal → 4, PrecisionGoal → 4]};
    maxLogLikeBlockCI[[d, year, farm, 2]] = {"p1", p1 /. maxLogLikeBlock[[
        d, year, farm, 2]], min /. FindRoot[CIfun0[min, z00[[d]]] == ml1,
        {min, 0.7 p1CIini, p1CIini}, AccuracyGoal → 4, PrecisionGoal → 4],
      max /. FindRoot[CIfun0[max, z00[[d]]] == ml1, {max, p1CIini, 1.5 * p1CIini},
        AccuracyGoal → 4, PrecisionGoal → 4]};
    , {d, {z0bestPos[[year, farm]]}}];
   ,
   Print["No significant distance"];
  ];
  , {farm, farms}];

(* Save MLL(block) *)
Clear[saveFile];
sFT = Flatten[Position[z0bestPos[[year]], _?Positive]];
saveFile = {Join[{"Farm"}, Table[farmTypes[[i]], {i, sFT}]],
  Join[{"z0"}, Table[z0best[[year, i]], {i, sFT}]],
```

```
  Join[{"p0"},
   Table[maxLogLikeBlock[[z0bestPos[[year, i]], year, i, 2, 1, 2]], {i, sFT}]],
  Join[{"p1"}, Table[maxLogLikeBlock[[z0bestPos[[year, i]],
     year, i, 2, 2, 2]], {i, sFT}]],
  Join[{"mll"}, Table[maxLogLikeBlock[[z0bestPos[[year, i]],
     year, i, 1]], {i, sFT}]],
  Join[{"llr"}, Table[logLikeRatio[[z0bestPos[[year, i]], year, i]],
    {i, sFT}]]};
Print[Column[{"Estimates of Block MLL in " <>
    ToString[dataYears[[year]]] <> ":", saveFile // Grid}]];
Export[FileNameJoin[{NotebookDirectory[], "results",
   ToString[dataYears[[year]]],
   dataYears[[year]] <> "-Block-MLL.txt"}], saveFile, "TSV"];
Clear[saveFile];
saveFile = {Join[{"Farm"}, Table[farmTypes[[i]], {i, sFT}]],
  Join[{"z0"}, Table[{z0min[[year, i]], z0max[[year, i]]}, {i, sFT}]],
  Join[{"p0"}, Table[{maxLogLikeBlockCI[[z0bestPos[[year, i]], year, i, 1, 3]],
     maxLogLikeBlockCI[[z0bestPos[[year, i]], year, i, 1, 4]]}, {i, sFT}]],
  Join[{"p1"}, Table[{maxLogLikeBlockCI[[z0bestPos[[year, i]], year, i, 2, 3]],
     maxLogLikeBlockCI[[z0bestPos[[year, i]], year, i, 2, 4]]}, {i, sFT}]]};
Print[Column[{"Confidence intervals of Block MLL in " <>
    ToString[dataYears[[year]]] <> ":", saveFile // Grid}]];
Export[FileNameJoin[{NotebookDirectory[], "results",
   ToString[dataYears[[year]]],
   dataYears[[year]] <> "-Block-CI.txt"}], saveFile, "TSV"];

(* BLOCK FUNCTION MAXIMUM LIKELIHOOD COMBINED
 FARM TYPES IN GIVEN YEAR (FIXED DISTANCE COMBINATIONS) *)

(* Get farm types with significant results *)
sFT = Flatten[Position[z0bestPos[[year]], _?Positive]];
(* Create empty table for all parameters to be explored *)
par = Table[{}, {year, Length[sFT] + 1}];
(* Create all possible distance combinations from z0best *)
zBestLists =  Tuples[DeleteDuplicates[z0best[[year, sFT]]], Length[sFT]];
(* for best fit only, substitute with {z0best[[year,sFT]]};*)
(* Perform joint analysis on each combination on z0best *)
Do[
 (* Get starting points for optimization from single farm type results *)
 kernelfun[0] := FindMinimum[((logLikeInf + logLikeEsc) /. Table[
      z0[i] → zBestLists[[zBL, Flatten[Position[sFT, i]][[1]]]], {i, sFT}]),
   Join[Table[{p0[i], maxLogLikeBlock[[z0bestPos[[year, i]], year, i,
        2, 1, 2]] * significantFarmTypes[[z0bestPos[[year, i]], year, i]]},
     {i, sFT}], {{p1, c00}}], AccuracyGoal → 4,
   PrecisionGoal → 4, Method -> "InteriorPoint"(*,
   StepMonitor:→Print[Join[Table[p0[i],{i,sFT}],{p1}]]*)];
 (* MLL *)
 Print["Calculating logLikeInf and logLikeEsc for ", dataYears[[year]],
  " data of patients in the vicinity of ", farmTypes[[sFT]], "..."];
 logLikeInf = Sum[pInfBlockMulti[i],
   {i, Transpose[Table[caseDistances[[farm]], {farm, sFT}]]}];
 logLikeEsc = Sum[pEscBlockMulti[i],
   {i, Transpose[Table[nonCaseDistances[[farm]], {farm, sFT}]]}];
```

```
Print["Calculating MLL(Block)Multi for ", dataYears[[year]],
 " data of patients in the vicinity of ", farmTypes[[sFT]],
 " at distance(s): ", ToString[zBestLists[[zBL]]]];
maxLogLikeBlockMulti[[year]] = Check[kernelfun[0], {}];
maxLogLikeBlockMulti[[year]] = If[NumericQ[
   maxLogLikeBlockMulti[[year, 1]]], maxLogLikeBlockMulti[[year]], {}];
If[maxLogLikeBlockMulti[[year]] ≠ {},
 Print["Calculating CI(Block)Multi for ", dataYears[[year]],
  " data of patients in the vicinity of ", farmTypes[[sFT]], "..."];
 (* Create the list of variables for CI calculations *)
 var = Join[Table[p0[i], {i, sFT}], {p1}];
 (* Get initial conditions for
  those variables from single farm type results *)
 ini = Join[Table[{p0[i], maxLogLikeBlockMulti[[year, 2,
       Flatten[Position[sFT, i]][[1]], 2]]}, {i, sFT}],
   {{p1, maxLogLikeBlockMulti[[year, 2, -1, 2]]}}];
 ml1 = maxLogLikeBlockMulti[[year, 1]] + 3.84;
 (* Calculate CIs *)
 Do[
  CIfun[p_?NumericQ] :=
   FindMinimum[((logLikeInf + logLikeEsc) /. Join[{var[[i]] → p}, Table[z0[j] →
          zBestLists[[zBL, Flatten[Position[sFT, j]][[1]]]], {j, sFT}]]),
     Select[ini, # ≠ ini[[i]] &], AccuracyGoal → 4, PrecisionGoal → 4,
     Method -> "InteriorPoint"][[1]];
  par[[i]] = {ToString[var[[i]]], var[[i]] /. maxLogLikeBlockMulti[[year, 2]],
    min /. FindRoot[CIfun[min] == ml1, {min, 0.7 ini[[i, 2]], ini[[i, 2]]},
      AccuracyGoal → 4, PrecisionGoal → 4],
    max /. FindRoot[CIfun[max] == ml1, {max, ini[[i, 2]], 1.5 * ini[[i, 2]]},
      AccuracyGoal → 4, PrecisionGoal → 4]};
  maxLogLikeBlockMultiCI[[year]] = par;
  Print[maxLogLikeBlockMultiCI[[year, i]]];
  , {i, Length[sFT] + 1}]
 ,
 Print["Empty MLL -> no CI calculation"]
];

Clear[saveFile];
sFT = Flatten[Position[z0bestPos[[year]], _?Positive]];
saveFile = {Join[{"Farm"}, Table[farmTypes[[i]], {i, sFT}]],
  Join[{"z0"},
   Table[zBestLists[[zBL, Flatten[Position[sFT, i]][[1]]]], {i, sFT}]],
  Join[{"p0"}, Table[maxLogLikeBlockMulti[[year, 2, i, 2]],
    {i, Length[sFT]}]],
  Join[{"p1"}, {maxLogLikeBlockMulti[[year, 2, Length[sFT] + 1, 2]]}],
  Join[{"mll"}, {maxLogLikeBlockMulti[[year, 1]]}]};
Print[Column[{"Estimates of Block MLL in " <>
    ToString[dataYears[[year]]] <> ":", saveFile // Grid}]];
Export[FileNameJoin[{NotebookDirectory[], "results", ToString[dataYears[[
     year]]], dataYears[[year]] <> "-BlockMultiSetDistance-MLL" <>
    ToString[zBestLists[[zBL]]] <> ".txt"}], saveFile, "TSV"];
Clear[saveFile];
saveFile = {Join[{"Farm"}, Table[farmTypes[[i]], {i, sFT}]],
  Join[{"z0"}, {{Min[z0min[[year, sFT]]], Max[z0max[[year, sFT ]]]}}],
```

```
      Join[{"p0"}, Table[{maxLogLikeBlockMultiCI[[year, i, 3]],
          maxLogLikeBlockMultiCI[[year, i, 4]]}, {i, Length[sFT]}]],
      Join[{"p1"}, {{maxLogLikeBlockMultiCI[[year, Length[sFT] + 1, 3]],
          maxLogLikeBlockMultiCI[[year, Length[sFT] + 1, 4]]}}]}];
    Print[Column[{"Confidence intervals of Block MLL in " <>
        ToString[dataYears[[year]]] <> ":", saveFile // Grid}]];
    Export[FileNameJoin[{NotebookDirectory[], "results", ToString[dataYears[[
          year]]], dataYears[[year]] <> "-BlockMultiSetDistance-CI" <>
        ToString[zBestLists[[zBL]]] <> ".txt"}], saveFile, "TSV"];

    , {zBL, Length[zBestLists]}];

   , {year, years}];

endSim = AbsoluteTime[];

Print["Execution speed: ", (endSim - startSim) / 60 , " min."];
```
